# Novel mutation of *SCN9A* gene causing generalized epilepsy with febrile seizures plus in a Chinese family

**DOI:** 10.1007/s10072-020-04284-x

**Published:** 2020-02-15

**Authors:** Tian Zhang, Mingwu Chen, Angang Zhu, Xiaoguang Zhang, Tao Fang

**Affiliations:** 1 Department of Pediatrics, The First Affiliated Hospital of USTC, Division of Life Sciences and Medicine, University of Science and Technology of China, Hefei, 230001 Anhui China; 2 Department of Pediatrics, Anhui Provincial Hospital Affiliated to Anhui Medical University, Hefei, 230001 Anhui China; 3 Department of Pediatrics, Anhui Provincial Hospital, Wannan Medical College, Wuhu, 241002 Anhui China

**Keywords:** SCN9A, Mutation, |Epilepsy, Generalized epilepsy with febrile seizures plus

## Abstract

Generalized epilepsy with febrile seizures plus (GEFS+) is a complex familial epilepsy syndrome. It is mainly caused by mutations in *SCN1A* gene, encoding type 1 voltage-gated sodium channel α-subunit (NaV1.1), and *GABRA1* gene, encoding the α1 subunit of the γ-aminobutyric acid type A (GABA_A_) receptor, while seldom related with *SCN9A* gene, encoding the voltage-gated sodium channel NaV1.7. In this study, we investigated a Chinese family with an autosomal dominant form of GEFS+. DNA sequencing of the whole coding region revealed a novel heterozygous nucleotide substitution (c.5873A>G) causing a missense mutation (p.Y1958C). This mutation was predicted to be deleterious by three different bioinformatics programs (The polyphen2, SIFT, and MutationTaster). Our finding reports a novel likely pathogenic *SCN9A* Y1958C heterozygous mutation in a Chinese family with GEFS+ and provides additional supports that *SCN9A* variants may be associated with human epilepsies.

**Electronic supplementary material:**

The online version of this article (10.1007/s10072-020-04284-x) contains supplementary material, which is available to authorized users.

## Introduction

*SCN9A* gene encodes the voltage-gated sodium channel NaV1.7, one of the nine known α members of voltage-gated sodium (Nav) channels [1]. It mainly expresses in dorsal root ganglion neurons, thus its mutations are mainly associated with pain disorders [2]. However, more and more studies have shown that *SCN9A* mutations in patients are also associated with variable epilepsy phenotypes including febrile seizures (FS) [3], GEFS+ [4], and Dravet syndrome (DS) [5] in recent years. *GABRG2* gene (γ-aminobutyric acid receptor subunit gene), *SCN1A* gene, and *GABRA1* gene are most frequently mentioned in GEFS+ [6–8], while the role of *SCN9A* gene in GEFS+ still remains unknown. Here, we report a novel previously unreported likely pathogenic *SCN9A* Y1958C heterozygous mutation with no *SCN1A* mutations in a Chinese family with GEFS+ and explore the possibility of *SCN9A* contributing to GEFS+.

## Material and methods

### Family recruitment

A family with 10 living Han members across three generation participated in the study. All the subjects signed the informed consent. Clinical data were collected from all members.

### Targeted exon capture and sequencing

After signing the consent, 2~4-ml peripheral blood from the ten members were collected. Blood Genome Column Medium Extraction Kit (Kangweishiji, China) was used to extract genomic DNA from blood samples. Whole-exome enrichment was performed using IDT_xGEN, which targets 39Mb protein-coding region of the human genome and covers 51Mb of partial intron. High-throughput sequencing was performed by Illumina NOVASeq 6000 series sequencer; the sequencing process was performed by Beijing Chigene Translational Medicine Research Center.

### Sequence alignment and variant calling

All reads were mapped to the human reference sequence (hg 19) using BWA-MEM (version 0.7.12). Then, a genome analysis tool kit (GATK version 3.4.0) was used to refine the alignments by performing local indel realignment and subsequent base quality recalibration. Single-nucleotide variants (SNVs) and insertions/deletions (indels) were called with the haplotype caller of the GATK. The variant was compared against publicly available databases such as the 1000 Genomes Project and the Exome Aggregation Consortium database (ExAC). At last, protein damage analysis was conducted to qualitatively predict the probability of the results by SIFT, PolyPhen-2, and MutationTaster, and multispecies alignments were performed using Mega 7.0 to determine whether the affected amino acids were conserved.

## Results

### Case description

**Fig. 1:**
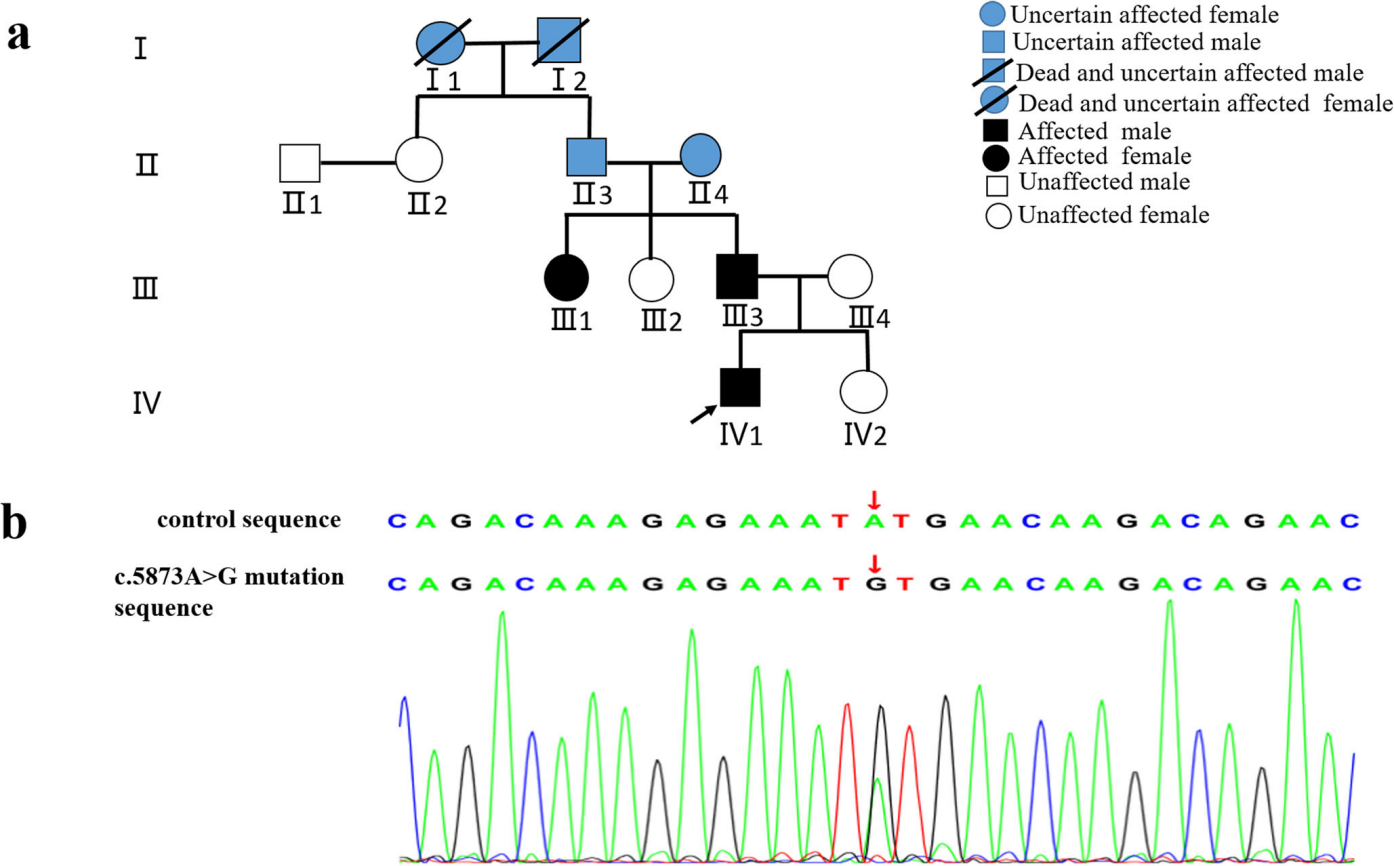
**a** Family pedigree. The black arrow indicates the proband; the legend for the symbols is at the right top of the figure. **b** Identification of a heterozygous mutation c.5873A>G (p.Y1958C) in the family members: proband (IV1), proband’s father (III3), proband’s aunt (III1), and proband’s grandmother (II4). Additional Sanger sequencing results are given in Online Resource (Figs. 3 and 4) The red arrow shows an A to G transition of nucleotide 5873

The proband (IV1) is a 9-year-old boy with normal spontaneous vaginal delivery and development. He presented the first seizure at 1 year and 4 months when encountering a respiratory tract infection with a fever (41 °C). Since then, he experienced febrile seizures for 9 times in total. The eighth seizure occurred at the age of 4, and he did not have convulsions in the next 4 years. The seizure patterns of the first time to the eighth time were all presented as generalized tonic-clonic seizures (GTCS), while the patient showed GTCS and absences on the ninth seizure when he was 9 years old with a fever. All the seizures last for about 1–2 min and could remit spontaneously. No obvious abnormality was found on neurological examination and brain magnetic resonance imaging (MRI). One week later after the last seizuring, electroencephalogram monitoring showed atypical spike-and-slow waves in the right temporal regions during sleep. In the further pedigree investigation, the proband’s father (III3) experienced febrile seizures at about 1 year old and one of the proband’s aunts (III1) reported febrile seizures at the age of two (Fig. 1a). The seizure patterns were also described as GTCS. Their seizures remitted spontaneously at that time and have not recurred until now. The proband’s grandmother (II4) and grandfather (II3) did not remember whether they had convulsions in their childhood. Besides, the family history of two dead family members (I1, I2) could not be obtained. The other family members denied seizure. None of them were treated with antiepileptic drugs for their seizures.

### Genetic findings

**Fig. 2:**
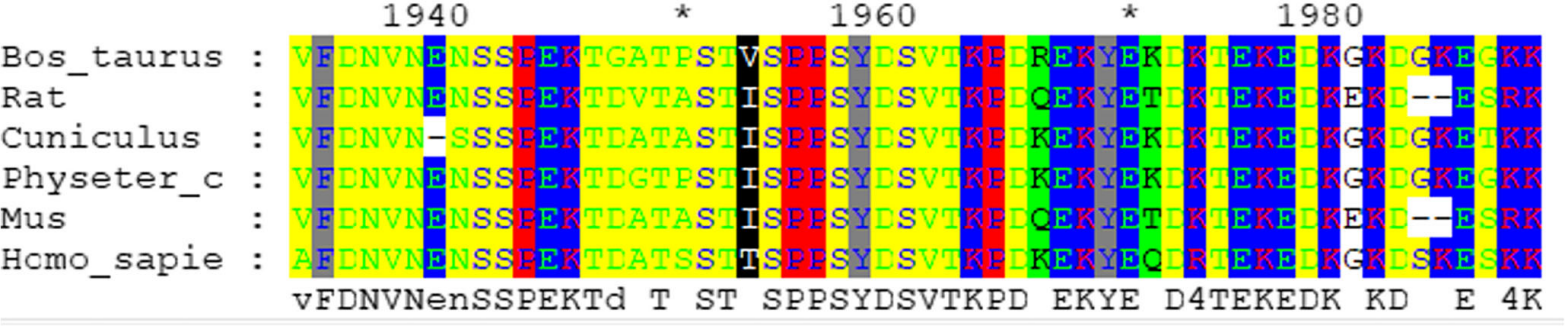
Alignment of multiple *SCN9A* protein sequences across species. The Y1958C affected amino acid locates in the highly conserved amino acid region in different mammals (from Ensembl). Red column shows the Y1958C site

A novel heterozygous *SCN9A* mutation (c.5873A>G) was detected in the proband (IV1), proband’s father (III3), proband’s aunt (III1), and proband’s grandmother (II4) by using clinical whole-exome sequencing, and Sanger sequencing was used to validate it (Fig. 1b). No mutations in *SCN1A* were detected. This mutation (c.5873A>G chr2:167055243 p.Y1958C) occurs in the population at a frequency of < 0.5% in the ExAC database (http://exac.broadinstitute.org/variant/2-167055243-T-C) and has not been reported in previous study or presented in dbSNP (http://evs.gs.washington.edu/EVS/) and 1000 Genomes Project (https://www.internationalgenome.org/). Multiple sequence alignment was performed by using Mega 7.0 (https://www.megasoftware.net/), and residue Y1958 is highly conserved (Fig. 2). The results of three bioinformatics programs show that the novel mutation could damage the function of the protein (Table 1). The pathogenicity of this variant is classified likely pathogenic, following the principle of standards and guidelines recommended by ACMG in recent publication [9].

**Table 1:** The prediction of the identified variant in *SCN9A*

Gene name	Position	Transcript	Substitution	ExAC	dbSNP ID	1000G	Polyphen2	SIFT	MutationTaster
*SCN9A*	chr2:167055243	NM_002977	c.5873A>G/Y1958C	0.0001	Novel	Novel	0.99 (probably damaging)	0.01 (damaging)	0.999993 (disease-causing)

*ExAC*, Exome Aggregation Consortium; *1000G*, 1000 genomes

## Discussion

GEFS+ is a complex autosomal dominant disorder with conspicuous phenotypic heterogeneity [10]. The first and second frequent phenotypes are febrile seizures, where generalized tonic-clonic seizures (GTCS) with fever occur between 3 months and 6 years, and febrile seizures plus (FS+), in which attacks with fever extend beyond 6 years or afebrile GTCS occur, respectively. The other phenotypes include FS/FS+ with absence, myoclonic, atonic, or focal seizures [11]. In our study, the proband experienced FS and FS+ with absence while his father and aunt only had febrile seizures, probably due to the incomplete penetrance and/or the phenotypic heterogeneity. As for the proband’s grandmother (II4), she did not remember whether convulsions had occurred in her childhood.

Several genes have been announced to be associated with GEFS+, in which *GABRG2*, *SCN1A*, and *GABRA1* account for the major part [6–8], while *SCN9A* is only reported in a few cases. A potentially pathogenic *SCN9A* variant, L266M (in exon 7), was discovered in one GEFS+ family [5]. Cen et al. reported a small pedigree diagnosed as GEFS+ with a heterozygous mutation (Q10R) in *SCN9A* gene without *SCN1A* mutation [4]. In 2019, a heterozygous mutation in the *SCN9A* gene, p.(Lys655Arg), in two sisters from a non-consanguineous family who presented GEFS+ was detected [11]. Actually, *SCN9A* variant is often mentioned as a genetic modifier in *SCN1A* mutation-associated epilepsy. A heterozygous *SCN9A* mutation, p.N641Y, was found to be responsible for a large Utah family (K4425) suffering from FS and GEFS+, and the authors identified nine *SCN1A* mutations with six different *SCN9A* mutations in this study [3]. However, the exact role of *SCN9A* mutations without *SCN1A* variants in GEFS+ has still remained left unclear. Thus, we hope to provide more evidence to illustrate that *SCN9A* has an important bearing on GEFS+ development in addition to these cases.

*SCN9A*, encoding sodium channel Nav1.7, contains 27 exons on chromosome 2q24.3 [12]. Nav1.7 is composed of 1977 amino acids and is organized into 4 domains, each with 6 trans-membranes [1]. In our study, a novel *SCN9A* heterozygous mutation (c.5873A>G) causing a missense mutation (p.Y1958C) was discovered. Regrettably, the mutation didn’t occur in the 4 and the exact cellular mechanism is unclear. However, the mutation in our study was located in highly conserved positions. The *SCN9A* N641Y mutation in FS proved to reduce thresholds to electrically induced seizures, and increase seizure susceptibility by targeted knock-in mouse model was also in highly conserved positions [3]. So, we speculate that the *SCN9A* Y1958C mutation might also affect the selectivity of the ion channel. Besides, the bioinformatics programs also demonstrated that the novel mutation could damage the function of the protein. All the evidences confirmed that the *SCN9A* p.Y1958C mutation should be regarded as pathogenic mutation in this family.

This report further supports that *SCN9A* mutation without *SCN1A* mutations is associated with GEFS+ and expands the spectrum of *SCN9A* gene, but there are limitations in our study that should be addressed. Thus, the functional effect of the mutation should be further studied to strengthen our views.

## Electronic supplementary material

ESM 1 (PNG 199 kb)

High resolution image (TIF 3098 kb)

ESM 2 (PNG 329 kb)

High resolution image (TIF 5032 kb)

## References

[1] Klugbauer N, Lacinova L, Flockerzi V, Hofmann F (1995). Structure and functional expression of a new member of the tetrodotoxin-sensitive voltage-activated sodium channel family from human neuroendocrine cells. EMBO J.

[2] Drenth JP, Waxman SG (2007). Mutations in sodium-channel gene SCN9A cause a spectrum of human genetic pain disorders. J Clin Invest.

[3] Singh NA, Pappas C, Dahle EJ, Claes LR, Pruess TH, De Jonghe P, Thompson J, Dixon M, Gurnett C, Peiffer A, White HS, Filloux F, Leppert MF (2009). A role of SCN9A in human epilepsies, as a cause of febrile seizures and as a potential modifier of Dravet syndrome. PLoS Genet.

[4] Cen Z, Lou Y, Guo Y, Wang J, Feng J (2017). Q10R mutation in SCN9A gene is associated with generalized epilepsy with febrile seizures plus. Seizure.

[5] Mulley JC, Bree H, Mcmahon JM, Xenia I, Susannah B, Mullen SA, Kevin F, Mark M, Lynette S, Andrew B (2014). Role of the sodium channel SCN9A in genetic epilepsy with febrile seizures plus and Dravet syndrome. Epilepsia.

[6] Audenaert D, Schwartz E, Claeys KG, Claes L, Deprez L, Suls A, Van Dyck T, Lagae L, Van Broeckhoven C, Macdonald RL, De Jonghe P (2006). A novel GABRG2 mutation associated with febrile seizures. Neurology.

[7] Wallace RH, Scheffer IE, Barnett S, Richards M, Dibbens L, Desai RR, Lerman-Sagie T, Lev D, Mazarib A, Brand N, Ben-Zeev B, Goikhman I, Singh R, Kremmidiotis G, Gardner A, Sutherland GR, George AL, Mulley JC, Berkovic SF (2001). Neuronal sodium-channel alpha1-subunit mutations in generalized epilepsy with febrile seizures plus. Am J Hum Genet.

[8] Johannesen K, Marini C, Pfeffer S, Møller RS, Maljevic S (2016) Phenotypic spectrum of GABRA1: from generalized epilepsies to severe epileptic encephalopathies. Neurology 87(11). 10.1212/WNL.0000000000003087 10.1212/WNL.0000000000003087 27521439

[9] Richards S, Aziz N, Bale S, Bick D, Das S, Gastier-Foster J, Grody WW, Hegde M, Lyon E, Spector E, Voelkerding K, Rehm HL, Comm ALQA (2015). Standards and guidelines for the interpretation of sequence variants: a joint consensus recommendation of the American College of Medical Genetics and Genomics and the Association for Molecular Pathology. Genet Med.

[10] Zhang YH, Burgess R, Malone JP, Glubb GC, Helbig KL, Vadlamudi L, Kivity S, Afawi Z, Bleasel A, Grattan-Smith P, Grinton BE, Bellows ST, Vears DF, Damiano JA, Goldberg-Stern H, Korczyn AD, Dibbens LM, Ruzzo EK, Hildebrand MS, Berkovic SF, Scheffer IE (2017). Genetic epilepsy with febrile seizures plus refining the spectrum. Neurology.

[11] Alves RM, Uva P, Veiga MF, Oppo M, Zschaber FCR, Porcu G, Porto HP, Persico I, Onano S, Cuccuru G, Atzeni R, Vieira LCN, Pires MVA, Cucca F, Toralles MBP, Angius A, Crisponi L (2019) Novel ANKRD11 gene mutation in an individual with a mild phenotype of KBG syndrome associated to a GEFS plus phenotypic spectrum: a case report. BMC Med Genet 20. 10.1186/s12881-019-0745-7 10.1186/s12881-019-0745-7 PMC6332862 30642272

[12] Ding JZJ, GUO Y, Zhang Y, Chen Z (2019). Novel mutations in SCN9A occurring with fever-associated seizures or epilepsy. Seizure.

