# Supplementary figures and images for: Novel mutation of SCN9A gene causing generalized epilepsy with febrile seizures plus in a Chinese family
Source: Neurol Sci. 2020 Feb 15;41(7):1913–7. doi: 10.1007/s10072-020-04284-x (PMC7359139; doi:10.1007/s10072-020-04284-x)

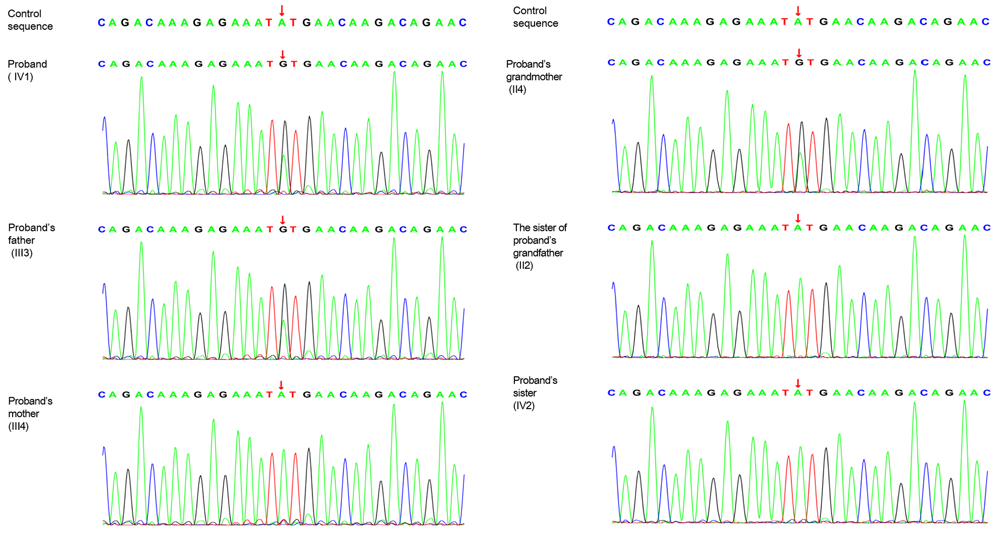

Supplement: Supplementary file 1 — (PNG 199 kb) [file 10072_2020_4284_Fig3_ESM.png]

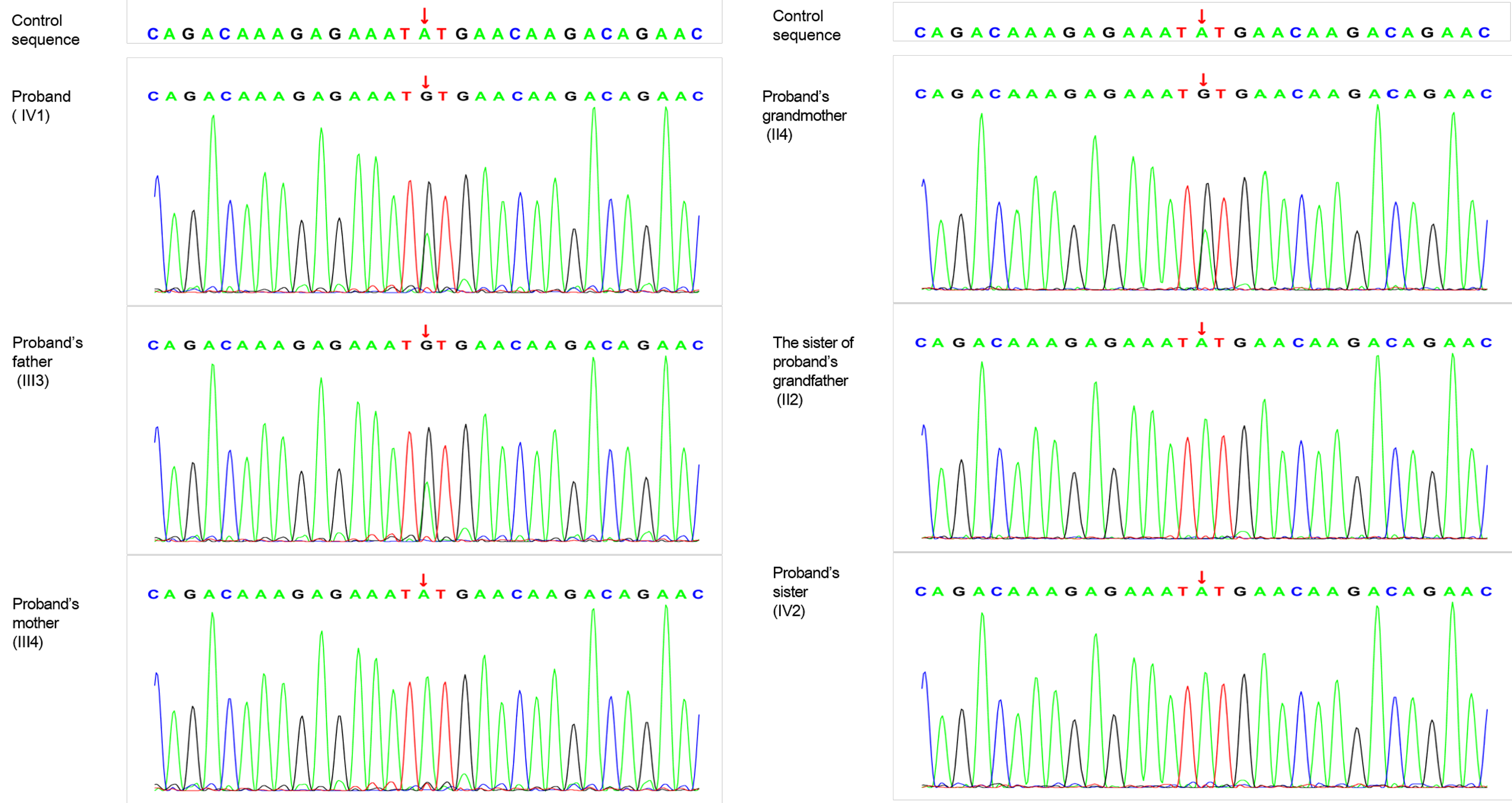

Supplement: Supplementary file 2 — High resolution image (TIF 3098 kb) [file 10072_2020_4284_MOESM1_ESM.tif]

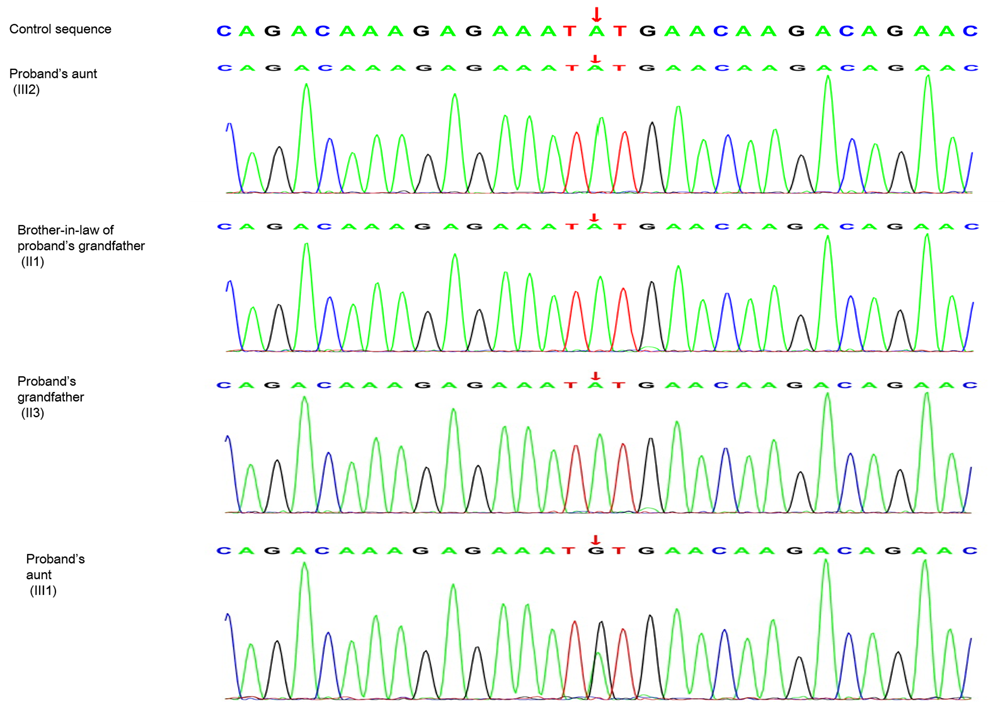

Supplement: Supplementary file 3 — (PNG 329 kb) [file 10072_2020_4284_Fig4_ESM.png]

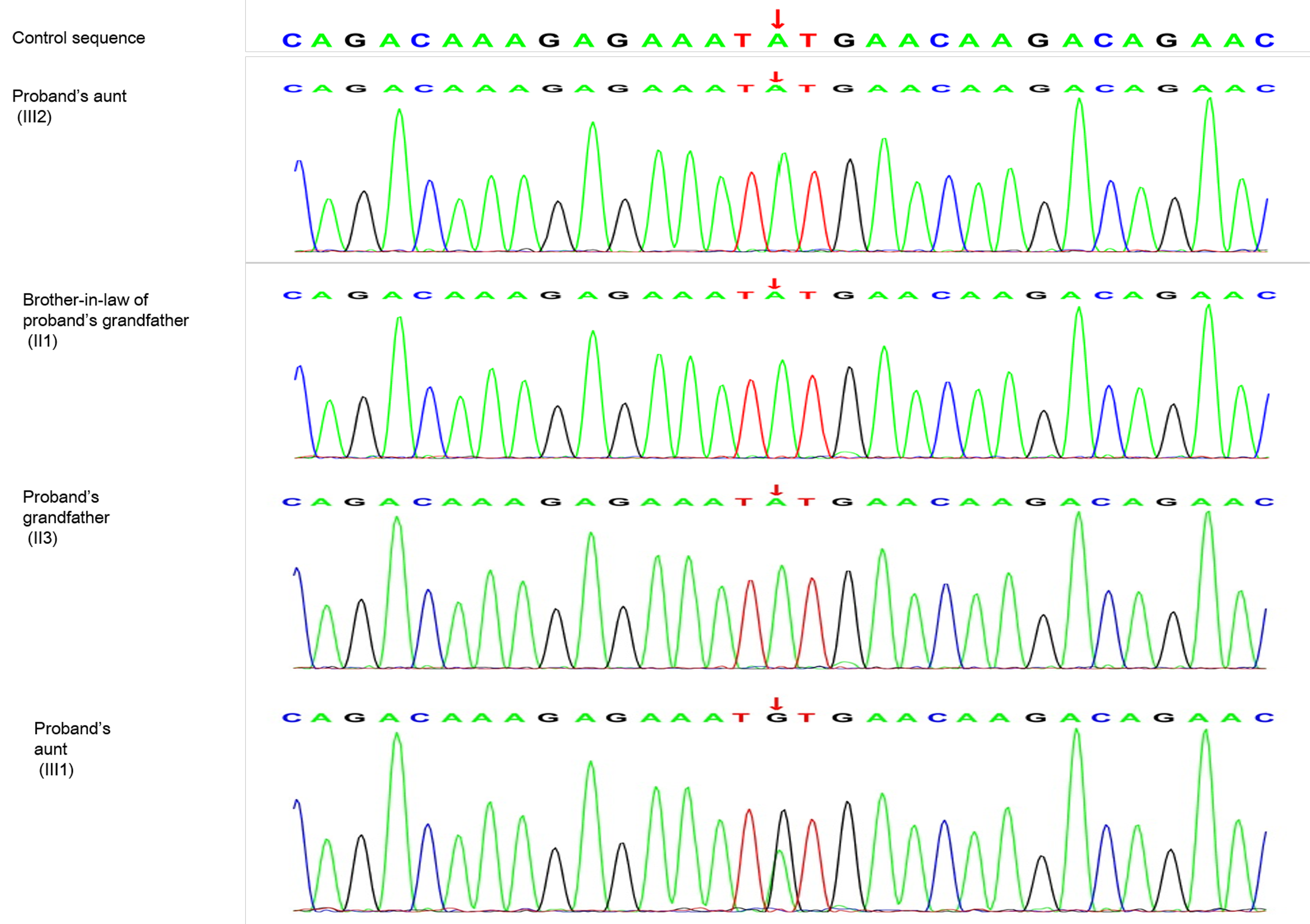

Supplement: Supplementary file 4 — High resolution image (TIF 5032 kb) [file 10072_2020_4284_MOESM2_ESM.tif]
